# Women’s knowledge on common non-communicable diseases and nutritional need during pregnancy in three townships of Ayeyarwaddy region, Myanmar: a cross-sectional study

**DOI:** 10.1186/s41182-019-0137-x

**Published:** 2019-01-29

**Authors:** Moe Moe Thandar, Junko Kiriya, Akira Shibanuma, Ken Ing Cherng Ong, Khaing Nwe Tin, Hla Hla Win, Masamine Jimba

**Affiliations:** 1 Department of Community and Global Health, Graduate School of Medicine, The University of Tokyo, Tokyo, Japan; 2 Maternal and Reproductive Health Division, Department of Public Health, Ministry of Health and Sports, Nay Pyi Taw, Myanmar; 3 Department of Preventive and Social Medicine, University of Medicine 1, Yangon, Myanmar

**Keywords:** Non-communicable disease, Nutrition, Knowledge, Pregnant women, Myanmar

## Abstract

**Background:**

Health systems in many countries do not adequately address non-communicable diseases (NCDs) during pregnancy, especially in low- and middle-income countries. In Myanmar, most studies on NCDs have investigated risk factors and prevalence of NCDs in the general population. This study aimed to assess the level of knowledge of common NCDs and nutritional need during pregnancy and to identify the factors associated with such knowledge, in three townships of Ayeyarwaddy region in Myanmar.

**Methods:**

A cross-sectional study was conducted among pregnant women aged between 18 and 49 years. We interviewed 630 pregnant women by using a pretested structured questionnaire. Knowledge questionnaire had five categories: general knowledge in NCDs, hypertension, diabetes, anemia, and nutritional need during pregnancy. Knowledge scores ranged from 0 to 56. We used Bloom’s cut-off point to classify the knowledge into three levels: low level as 59% or below (0–33 scores), moderate level as 60–80% (34–49 scores), and high level as 80–100% (50–56 scores). We conducted multiple linear regression analyses to find the association between different exposure variables (behavioral risk factors, pre-existing NCDs in pregnant women, and family history of NCDs) and knowledge on common NCDs and nutritional need during pregnancy adjusted for socioeconomic factors.

**Results:**

Among pregnant women, 64.8% had moderate level of knowledge, 22.7% had low level, and only 12.5% had high level. The mean knowledge scores were 39.6 (standard deviation 10.2). Pregnant women with the following factors were more likely to have higher knowledge: their belonging to the second, middle, and fourth quintiles of wealth index and their family members’ status of having some kind of NCDs.

**Conclusions:**

Majority of pregnant women had low to moderate level of knowledge on common NCDs and nutritional need during pregnancy. Wealth and family history of NCDs were significantly associated with their knowledge. Prevention and promotion of NCDs should be integrated in maternal and child health programs and should emphasize for the pregnant women who are in the poorest or richest wealth quintiles and who do not have family history of NCDs.

## Background

Indirect causes of maternal death are becoming prominent and accounting for more than a quarter of maternal deaths worldwide [1]. Indirect causes include deaths due to communicable diseases, non-communicable diseases (NCDs), and other indirect causes such as accidents [2, 3]. Emphasis has been placed on communicable disease during pregnancy such as prevention and control of malaria among pregnant women. However, health systems in many countries do not adequately address NCDs during pregnancy, especially in low- and middle-income countries (LMIC).

Important NCDs during pregnancy include a large number of different medical conditions. They are cardiovascular diseases such as hypertension, endocrine, or metabolic diseases such as diabetes, hematological diseases such as anemia, mental illness such as depression, and neoplasm [4]. NCDs during pregnancy have a significant adverse effect on maternal health and pregnancy outcomes. Hypertension, diabetes, anemia, obesity, overweight, and undernourishment during pregnancy are linked to hemorrhage, pre-eclampsia, stillbirth, low birth weight, preterm birth, congenital malformation, and maternal and neonatal mortality [5–8].

Non-communicable diseases are increasingly affecting LMIC, and about 78% of NCD-related deaths occurred in LMIC [9]. Myanmar is one of the LMIC in Southeast Asia (SEA) with the lowest life expectancy and the second-highest rate of maternal and child mortality [10–13]. To reduce maternal and child mortality, Myanmar placed a lot of inputs in Maternal and Child Health (MCH) services in both urban and rural areas [14]. The core elements of MCH services in Myanmar are pregnancy, delivery, postnatal, and newborn care; birth spacing and family planning; miscarriage and post-abortion care; adolescent and youth reproductive health; and screening and treatment of sexually transmitted infections and cervical cancer [15].

In 2014, the World Health Organization (WHO) estimated that NCDs account for 59% of deaths in Myanmar [16]. In Myanmar, 27% of maternal deaths in 2016 were due to indirect causes [17]. According to 2014 WHO STEPS survey in Myanmar, women were at higher risk of NCDs than men as a result of low consumption of fruits and vegetables, insufficient physical activity, overweight and obesity, high blood pressure, high blood sugar, and raised blood cholesterol [18]. Myanmar Demographic and Health Survey (DHS) also reported that 47% of women aged between 15 and 49 years were anemic in 2015 [19]. Myanmar is now prioritizing NCDs in its National Health Plan and started to expend Package of Essential Non-communicable Diseases Intervention (PEN) for treatment and referral of NCDs in the primary health care [20, 21].

Most studies on NCDs have focused on risk factors and prevalence of NCDs among general population in Myanmar [22–26]. Limited studies are available on knowledge on common NCDs and nutritional need during pregnancy among women in Myanmar. This study aimed to assess the level of knowledge of common NCDs and nutritional need during pregnancy and to identify the factors associated with such knowledge among women in three townships of Ayeyarwaddy region in Myanmar.

## Methods

### Study design and setting

We conducted this cross-sectional study as a baseline study of a cluster randomized controlled trial (RCT). This cluster RCT has two objectives: the first one is to test the effect of a continuum of care (CoC) card on mothers’ utilization of CoC services, and the second one is to test if integration of health education on common NCDs and nutritional need during pregnancy in antenatal and postnatal care services could increase mothers’ knowledge on common NCDs and nutritional need during pregnancy [27]. The unit of randomization is primary health center, particularly MCH Center in urban area and Rural Health Center (RHC) in rural area. We conducted this study in Pantanaw, Wakema, and Ingapu townships in the Ayeyarwaddy region of Myanmar. These three townships are located in predominantly rural area. We included all health centers in both rural and urban areas, 3 MCH centers and 29 RHCs.

### Participants

The eligible participants were pregnant women who received the antenatal care (ANC) from health centers. Inclusion criteria were pregnant women who aged between 18 and 49 years and were between 12 and 20 weeks of pregnancy when they received the first ANC. We excluded pregnant women if they received ANC more than once by the time of recruitment, because we would like to know if the CoC card could encourage them to receive follow-up visits. We also excluded pregnant women who were migrants or mobile populations. In this study, we defined migrant as residents for more than 6 months and less than 1 year and mobile population as residents for less than 6 months in a specific area, according to Myanmar Population and Housing Census, 2015 [28].

### Sample size

We calculated the sample size for the cluster RCT based on a formative study on CoC completion among mothers in two townships of Myanmar in 2015. By using an intraclass correlation coefficient of 0.2, the confidence interval (CI) of 95%, the power of 90%, and potential attrition of 15%, we estimated 640 pregnant women would be required.

### Sampling procedure

We used a multi-stage sampling process. First, we purposely selected Ayeyarwaddy region as it was one of the top three regions in terms of maternal mortality in Myanmar [29]. Ayeyarwaddy region consists of six districts, which in turn are composed of 33 townships [30]. Second, we purposely selected Pantanaw township from Maubin district, Wakema township from Myaungmya district, and Ingapu township from Hinthada district because they have similar sociodemographic background. Finally, we included all health centers in three townships and obtained the list of pregnant women who received ANC in all health centers. Of 1384 eligible pregnant women, 804 met inclusion criteria. We visited their households and recruited 630 pregnant women (222 from Pantanaw, 172 from Wakema, and 236 from Ingapu townships) in the baseline study. The response rate was 78.4% as 174 pregnant women were absent at the time of recruitment.

### Study instrument

We developed a structured questionnaire based on relevant previous studies [31–37]. We first developed the questionnaire in English, made a forward translation into Burmese, and back-translated it into English. The questionnaire included socioeconomic information, behavioral risk factors for NCDs, history of NCDs in pregnant women, family history of NCDs, and knowledge on common NCDs and nutritional need during pregnancy. Before the survey, we conducted pretests with 30 women to improve the readability and to validate the content of questionnaire. The Cronbach’s alpha of the knowledge questions in this study was 0.85.

Socioeconomic information included age, education, occupation, ethnicity and religion, and wealth index. We measured wealth index by using a household asset index. To calculate an asset index, we conducted a principal component analysis by using information on water source, electricity source, toilet type, and household assets including radio, television, computer, mobile telephone, fridge, car, motorbike, bicycle, washing machine, and gas or electric cooker.

Behavioral risk factors included their smoking, betel chewing, and alcohol drinking during pregnancy and also their family members’ smoking status during their pregnancy. For history of NCDs in pregnant women, we asked if they were ever diagnosed with hypertension, cardiovascular disease, diabetes, cancer, and chronic respiratory diseases before pregnancy. For their family history of NCDs, we asked them if their blood-related family members had above diseases.

We developed knowledge questions by mainly considering three common NCDs (hypertension, diabetes, and anemia) and nutrition need during pregnancy. Knowledge questions composed of five categories: (1) general knowledge in NCDs, (2) hypertension, (3) diabetes, (4) anemia, and (5) nutritional need during pregnancy. General knowledge in NCDs contained the knowledge about behavioral risk factors for NCDs, the most common cause of death among women, and prevention for NCDs. For hypertension, diabetes, and anemia, we assessed their knowledge about their causes, symptoms, preventions, treatments, and complications. For nutritional need during pregnancy, we asked their knowledge about the food groups, recommended food intake, and complications of undernourishment during pregnancy. We used close-ended questions with predefined choices, for example, “Is hypertension a curative disease” with response choices of “Yes,” “No,” and “Do not know.” We scored 1 for each correct answer and 0 for each incorrect or “Do not know” answer. Therefore, the knowledge score for 56 questions ranged from 0 to 56.

### Variables

The dependent variable was pregnant women’s knowledge scores on common NCDs and nutritional need during pregnancy. The independent variables were 1) socio-economic characteristics, 2) behavioral risk factors for NCDs, 3) pre-existing NCDs in pregnant women and 4) family history of NCDs.

We asked the highest education they completed and categorized education level as follows: (1) no education (those who did not go to school, (2) primary school, (3) middle school, and (4) high school or university. We categorized the ethnicity as Bamar and others (Kachin, Kayin, and Muslim) and the religion as Buddhist and others (Christian and Islam). We divided the household asset index into five quintiles of wealth index: (1) the lowest quintile, (2) the second quintile, (3) the middle quintile, (4) the fourth quintile, and (5) the highest quintile. For behavioral risk factors, we considered as “presence” if they reported any risk factor. For NCDs, we considered as “presence” if they reported any kind of NCDs. Similarly, we considered as “presence” for family history of NCDs if they reported any kind of NCDs in their family members.

### Data collection

We conducted face-to-face interviews in May and June 2017. We collected data with android mobile devices by using Open Data Kit technology developed by the researchers at the University of Washington’s Department of Computer Science and Engineering.

### Data analysis

We used descriptive statistics to present the exposure variables and the accuracy rate for each knowledge question. We used Bloom’s cut-off point to classify the knowledge into three levels as follows: low level as 59% or below (0–33 scores), moderate level as 60–80% (34–49 scores), and high level as 80–100% (50–56 scores) [38–41]. We performed simple and multiple linear regression analyses by using the variables that were found to be associated with NCD-related knowledge in previous studies [36, 42–45]. In model 1, we included behavioral risk factors and socioeconomic characteristics. In model 2, we included the pre-existing NCDs in pregnant women and socioeconomic characteristics. In model 3, we included family history of NCDs and socioeconomic characteristics. In the final model, we included behavioral risk factors, pre-existing NCDs in pregnant women, and family history of NCDs adjusting for socioeconomic characteristics. We set the level of significance at *p* < 0.05. We used STATA 13.1 (StataCorp LP, College Station, TX, USA) for all data analysis.

## Results

Table 1 shows the socioeconomic characteristics of participants. Of the 630 pregnant women, 53.5% were aged between 20 and 29 years, 47.5% completed primary school, and 45.4% were housewives. Majority of them were Bamar ethnicity (66.8%) and Buddhist (93.5%).

**Table 1:** Socio-economic characteristics of pregnant women (*n* = 630)

	Number	Percent
Age (years)		
18–19	49	7.8
20–29	337	53.5
30–39	220	34.9
40–49	24	3.8
Education		
No schooling	44	7.0
Primary school completed	299	47.5
Middle school completed	142	22.5
High school completed or graduated	145	23.1
Occupation		
Housewife	286	45.4
Farmer	135	21.4
Others	209	33.2
Ethnic		
Bamar	421	66.8
Others	209	33.2
Religion		
Buddhist	589	93.5
Others	41	6.5
Gravida		
Primigravida	279	44.3
Multigravida	351	55.7
Wealth index		
Lowest quintile	126	20.0
Second quintile	127	20.2
Middle quintile	131	20.8
Fourth quintile	122	19.4
Highest quintile	124	19.7

**Fig. 1:**
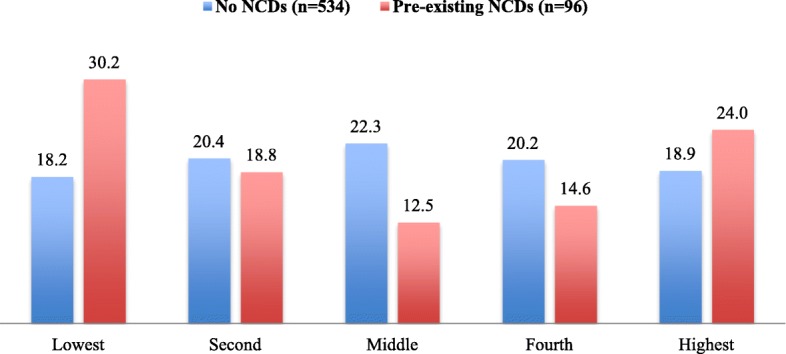
Prevalence of pre-existing NCDs among pregnant women with different wealth quintiles (*n* = 630)

Table 2 shows medical history of pregnant women. Among 630 pregnant women, 12 of them smoked, 86 women had betel chewing habit, and 8 women reported alcohol drinking during the pregnancy. In addition, 274 women (43.5%) reported that someone in their family was smoking during the pregnancy. Hypertension was the most common reported disease among them (6.7%) and in their blood-related family members (35.1%). Among women who reported any kind of NCDs (*n* = 96), the rate was the highest in the lowest quintile (30.2%) followed by the highest quintile (24.0%) (Fig. 1).

**Table 2:** Medical history of pregnant women (*n* = 630)

	Number	Percent
Behavioral risk factors		
Smoking	12	1.9
Betel chewing	86	13.7
Alcohol drinking	8	1.3
Smoking in family members	274	43.5
Pre-existing NCDs in pregnant women		
Hypertension	42	6.7
Diabetes	4	0.6
Cardiovascular diseases	39	6.2
Cancer	1	0.2
COPD	25	4.0
Family history of NCDs		
Hypertension	221	35.1
Diabetes	102	16.2
Cardiovascular diseases	97	15.4
Cancer	71	11.3
COPD	103	16.4

Table 3 shows the accuracy rate of response to each knowledge question. The accuracy rate for the question, “The most common cause of death among women around the world”, was the lowest (16.2%) among all the questions. Regarding the risk factors for NCDs, the accuracy rate for tobacco use, harmful alcohol consumption, and physical inactivity was less than 40%. Few pregnant women knew that hypertension and diabetes were not curable (accuracy rate of 19.4% in hypertension and 28.3% in diabetes). The well-known risk factors for hypertension were old age (80.0%), obesity (83.2%), consuming salty food (95.7%), and alcohol drinking (77.5%) while less well-known risk factors were family history of hypertension (55.6%) and smoking (56.0%). Similarly, the well-known risk factors for diabetes were old age (78.7%), obesity (83.4%), and consuming sweet, fried, and fatty food (92.5%) while less well-known risk factors for diabetes were family history of diabetes (60.5%) and pregnancy (51.4%). In terms of anemia, pregnant women exhibited a satisfactory understanding of the causes, symptoms, and complications of anemia (accuracy rates from 78.4 to 94.3%) and iron-rich food for anemia prevention (more than 80%). However, about 60% of them knew that vitamin C enhance the iron absorption and about 53% knew that caffeine in tea or coffee disturb the iron absorption. Regarding nutritional need during pregnancy, more than 90% of them knew that they should not abstain food during pregnancy, they should eat more especially in the second and third trimesters, and they should eat nutritious food to prevent adverse pregnancy outcomes. However, they did not know about three major food groups (accuracy rate of 20.8% in carbohydrates, 40.0% in protein, and 50.6% in vitamins and minerals).

**Table 3:** Accuracy rate of response to each knowledge question

	Number	Percent
General knowledge in NCDs		
Behavior risk factors for developing non-communicable diseases		
Tobacco use	235	37.3
Harmful alcohol consumption	244	38.7
Unhealthy diet	426	67.6
Physical inactivity	251	39.8
The most common cause of death among women around the world		
Cardiovascular disease	102	16.2
The good way to prevent non-communicable diseases		
Eating more fruits and vegetables	540	85.7
Hypertension		
Hypertension is a curative disease.	122	19.4
Blood pressure of 140/90 mmHg is considered high.	441	70.0
Common symptoms of hypertension are headache, dizziness, blurred vision, nausea, vomiting, and fainting attack.	540	85.7
Elderly persons are more susceptible to hypertension than adults.	504	80.0
If your blood-related relatives or family members have hypertension, you are also at risk of hypertension.	350	55.6
Obese people are more at risk of hypertension than those who are not obese.	524	83.2
Smoking increases risk for having hypertension.	353	56.0
Consuming salty food increases risk for having hypertension.	603	95.7
Alcohol drinkers are at risk of having hypertension.	488	77.5
Regular exercisers are less likely to have hypertension than those who do not exercise	507	80.5
Complications of hypertension		
Stroke	495	78.6
Coronary artery disease	446	70.8
Heart failure	447	71.0
Blindness	397	63.0
Renal failure	366	58.1
Diabetes		
Diabetes is a curative disease.	178	28.3
Suspicious diabetes symptoms are frequent urination and frequent water drinking from thirst.	471	74.8
Elderly persons are more susceptible or more likely to have diabetes than adults.	496	78.7
If your blood-related relatives have diabetes, you are also at risk of having diabetes.	381	60.5
Obese people are more at risk of diabetes than those who are not obese.	519	82.4
Pregnant women are likely to have diabetes.	324	51.4
People who regularly eat sweet, fried, and fatty food are at risk of having diabetes.	583	92.5
Regular exercisers are less likely to have diabetes than those who do not exercise.	496	78.7
Complications of diabetes		
Blindness	427	67.8
Renal failure	429	68.1
Heart failure	411	65.2
Stroke	434	68.9
Delay ulcer healing	531	84.3
Hypertension	476	75.6
Anemia		
Iron deficiency can cause anemia.	494	78.4
Anemia can be found in both male and female at any age.	569	90.3
If a pregnant woman has anemia, she can deliver a low birth weight baby.	517	82.1
Symptoms of anemia		
Paleness on skin, eye and lips	561	89.1
Weakness	586	93.0
Headache	594	94.3
Shortness of breath	571	90.6
Rapid heart rate	551	87.5
Iron-rich food		
Leafy green vegetables	549	87.1
Beans and peas	532	84.4
Meat and fish	523	83.0
Eating vitamin C-rich food along with vegetables and beans can help your body to easily absorb iron.	380	60.3
Drinking tea and coffee after meal can disturb the iron absorption.	331	52.5
Nutrition		
Please mention three food groups		
Carbohydrates	131	20.8
Proteins	252	40.0
Vitamins and minerals	319	50.6
Pregnant woman should not avoid certain food such as beans, vegetables and meat.	609	96.7
Pregnant woman should eat more especially second (13–27 weeks) and third trimester (28–36 weeks).	595	94.4
If pregnant woman does not eat nutritious food, she has risk of maternal and child death.	603	95.7
Malnutrition during pregnancy can contribute to low birth weight and preterm delivery	606	96.2
Malnutrition during pregnancy can contribute to miscarriage and stillbirth	591	93.8

Table 4 shows the distribution of knowledge levels among pregnant women. Among them, 64.8% had moderate level of knowledge, 22.7% had low level of knowledge, and only 12.5% had high level of knowledge. The mean knowledge score was 39.6 (standard deviation 10.2).

**Table 4:** Distribution of level of knowledge (*n* = 630)

Level	Number	Percent
Low (0–33 scores)	143	22.7
Moderate (34–49 scores)	408	64.8
High (50–56 scores)	79	12.5

Minimum = 0, maximum = 56, mean = 39.6, SD = 10.2

Table 5 demonstrates the results of simple and multiple linear regression analyses. Age, education, wealth index, and family history of NCDs were associated with knowledge in simple linear regression. According to the final multiple linear regression model, only wealth index and family history of NCDs were associated with their knowledge. Women belonging to the second (*B* = 3.5, *p* = 0.007), middle (*B* = 3.5, *p* = 0.08), and fourth (*B* = 3.2, *p* = 0.019) quintiles of wealth index had higher knowledge than the lowest quintile. Women whose family member had some kind of NCDs had higher knowledge (*B* = 1.8, *p* = 0.028) compared to the referenced group.

**Table 5:** Factors associated with knowledge on common NCDs and nutritional need during pregnancy (*n* = 630)

	Simple linear regression				Model I (behavioral risk factors and knowledge)				Model II (pre-existing NCDs and knowledge)				Model III (family history of NCDs and knowledge)				Model IV (final)			
	Coef.	*p* value	95%CI		Coef.	*p* value	95%CI		Coef.	*p* value	95%CI		Coef.	*p* value	95%CI		Coef.	*p* value	95%CI	
Women’s age																				
≤ 19 (ref)																				
20–29	2.3	0.143	− 0.8	5.3	1.2	0.451	− 1.9	4.3	1.3	0.419	− 1.8	4.3	1.0	0.519	− 2.1	4.1	1.0	0.542	− 2.1	4.0
30–29	4.2	*0.009*	1.1	7.4	3.2	0.051	0.0	6.3	3.1	0.053	0.0	6.3	2.9	0.069	− 0.2	6.1	3.0	0.063	− 0.2	6.2
40–49	4.0	0.114	− 1.0	8.9	3.7	0.146	− 1.3	8.7	3.6	0.152	− 1.3	8.6	3.7	0.141	− 1.2	8.7	3.8	0.131	− 1.1	8.8
Ethnic																				
Bamar (ref)																				
Other	− 0.6	0.494	− 2.3	1.1	− 0.7	0.396	− 2.4	1.0	− 0.5	0.531	− 2.2	1.1	− 0.6	0.506	− 2.3	1.1	− 0.8	0.357	− 2.5	0.9
Education																				
Didn’t go to school (ref)																				
Primary school	2.7	0.097	− 0.5	5.9	2.0	0.214	− 1.2	5.3	1.9	0.241	− 1.3	5.2	2.2	0.184	− 1.0	5.4	2.0	0.234	− 1.3	5.2
Middle school	3.3	0.061	− 0.2	6.7	2.4	0.185	− 1.1	5.9	2.1	0.241	− 1.4	5.6	2.4	0.177	− 1.1	5.9	2.2	0.222	− 1.3	5.7
High school	4.2	*0.016*	0.8	7.6	3.3	0.077	− 0.4	6.9	3.2	0.085	− 0.4	6.8	3.5	0.057	− 0.1	7.1	3.1	0.091	− 0.5	6.7
Occupation																				
Housewife (ref)																				
Farmer	1.4	0.174	− 0.6	3.5	1.1	0.311	− 1.0	3.2	1.1	0.286	− 1.0	3.3	1.1	0.294	− 1.0	3.2	1.0	0.346	− 1.1	3.1
Others	0.3	0.726	− 1.5	2.1	0.2	0.793	− 1.6	2.1	0.2	0.810	− 1.6	2.1	0.2	0.812	− 1.6	2.0	0.3	0.771	− 1.6	2.1
Wealth_index2																				
Lowest (ref)																				
Second	3.9	*0.002*	1.4	6.4	3.3	*0.010*	0.8	5.8	3.5	*0.006*	1.0	6.1	3.4	*0.008*	0.9	5.9	3.5	*0.007*	1.0	6.0
Middle	4.1	*0.001*	1.6	6.6	3.3	*0.012*	0.7	5.8	3.7	*0.006*	1.1	6.2	3.3	*0.011*	0.8	5.9	3.5	*0.008*	0.9	6.1
Fourth	3.9	*0.003*	1.4	6.4	3.1	*0.024*	0.4	5.7	3.4	*0.014*	0.7	6.0	3.0	*0.028*	0.3	5.6	3.2	*0.019*	0.5	5.8
Highest	3.1	*0.017*	0.6	5.6	1.9	0.168	− 0.8	4.7	2.1	0.136	− 0.7	4.9	1.8	0.209	− 1.0	4.5	1.9	0.181	− 0.9	4.6
Behavioral risk factors	− 1.5	0.061	− 3.1	0.1	− 1.6	0.057	− 3.2	0.0									− 1.5	0.070	− 3.1	0.1
Pre-existing NCDs	1.6	0.145	−0.6	3.9					2.1	0.067	− 0.1	4.3					1.4	0.221	− 0.9	3.7
Family history of NCDs	2.1	*0.012*	0.5	3.7									2.1	*0.012*	0.5	3.6	1.8	*0.028*	0.2	3.5
*R*^2^					0.05				0.05				0.05				0.06			
Adjusted *R*^2^					0.03				0.03				0.03				0.04			

## Discussion

In this study, the majority of pregnant women had low to moderate level of knowledge on common NCDs and nutritional need during pregnancy. Factors associated with their knowledge scores were wealth index and family history of NCDs.

About 85% of pregnant women in this study had low to moderate level of knowledge on common NCDs and nutritional need during pregnancy. Regarding behavioral risk factors for NCDs, less than 40% of them did not know that smoking, harmful alcohol consumption, and physical inactivity were their risk factors for NCDs. Majority of pregnant women in this study came from rural areas, and they did not notice that smoking and alcohol consumption as risk factors because these were common in their society. Therefore, among behavioral risk factors for NCDs, smoking and alcohol consumption were the most commonly reported behavioral risk factors in rural Myanmar [23].

Regarding hypertension and diabetes, about one fifth of them thought that hypertension and diabetes were curable. This finding was different from the study done among Karen ethnic high school students in Thai-Myanmar border area. In that study, more than two thirds of students realized that hypertension was not curable and about 30% of them recognized diabetes as incurable disease [31]. Majority of pregnant women knew that old age, obesity, and salty or sweet, fried, and fatty food were risk factors for hypertension and diabetes. However, only half of them knew that family history was risk factor for both diseases. Previous studies showed that family history was less recognized as one of the causes of hypertension [46, 47].

The accuracy rates for the questions about anemia and nutritional need during pregnancy were high compared to those in general knowledge in NCDs, hypertension, and diabetes. Majority of pregnant women knew the causes, symptoms, and complications of anemia; iron-rich food; and complications of malnutrition during pregnancy. This may be because anemia and nutrition education has been provided during ANC visits in Myanmar [15] while other NCD-related health education was not compulsorily provided to them.

Wealth index was associated with their knowledge scores. Pregnant women belonging to the second, middle, and fourth quintiles of wealth index had higher knowledge scores compared to those in the lowest and highest quintiles. The poorest and the richest have the risky health behaviors such as stress, unbalance diet, and physical inactivity [48–50]. In this study, the rates of pre-existing NCDs among pregnant women were higher in the lowest and highest quintiles compared to the three middle quintiles. This may be due to concentration of risky behaviors and lower knowledge among the poorest and the richest.

Furthermore, having family history of NCDs was associated with the pregnant women’s higher knowledge scores. This may be due to their involvement in family members’ NCD management. Family members of diabetes mellitus patients, for example, had higher knowledge on diabetes than others [32, 51–53].

This study has some limitations. First, it was a cross-sectional study and there was a chance of reverse causality [54]. Having history of NCDs and smokers in family members might be the results rather than the causes of higher level of knowledge. Second, this study used a newly developed instrument in Burmese. Although the questions were pretested for reliability, the questionnaire might have been comparatively easy to choose correct answers. Third, self-reported medical and family history of NCDs may not be completely valid, as we did not examine their health records. And we did not ask history of anemia in pregnant women and in their family members. Fourth, this study did not explore the knowledge on other important NCDs such as cancer and chronic respiratory diseases. Fifth, this study was conducted in predominantly rural areas. Therefore, the findings may not reflect the urban area.

Despite such limitations, this study owns many strengths. To the best of our knowledge, this is the first study to access the knowledge on common NCDs and nutritional need during pregnancy among women in Myanmar and other LMIC in SEA. This study also identified the family factor associated with their knowledge on common NCDs and nutritional needs during pregnancy. The finding from this study is applicable in designing health education programs for pregnant women, especially in rural areas, in other LMIC in SEA.

## Conclusion

Majority of pregnant women had low to moderate level of knowledge of common NCDs and nutritional need during pregnancy. Wealth and family history of NCDs were significantly associated with their knowledge. Prevention and promotion of NCDs should be integrated in maternal and child health programs and should emphasize for the pregnant women who are in the poorest or richest wealth quintiles and who do have family history of NCDs.

## Abbreviations

ANC: Antenatal care
CI: Confidence interval
CoC: Continuum of care
DHS: Demographic and Health Surveillance
LMIC: Low- and middle-income country
MCH: Maternal and Child Health
NCD: Non-communicable diseases
PEN: Package of Essential Non-communicable Diseases Intervention
RCT: Randomized control trial
SEA: Southeast Asia
STEPS: STEPwise approach to non-communicable disease surveillance
WHO: World Health Organization
